# 15-Lipoxygenase-1 Is Involved in the Effects of Atorvastatin on Endothelial Dysfunction

**DOI:** 10.1155/2016/6769032

**Published:** 2016-08-10

**Authors:** Peng Zhang, Xin Xing, Chunxiao Hu, Hui Yu, Qian Dong, Guanglei Chang, Shu Qin, Jian Liu, Dongying Zhang

**Affiliations:** Department of Cardiology, The First Affiliated Hospital of Chongqing Medical University, No. 1 Yixueyuan Road, Yuzhong District, Chongqing 400016, China

## Abstract

Statins exert pleiotropic effects on endothelial cells in addition to lowering cholesterol. 15-Lipoxygenase-1 (ALOX15) has been implicated in vascular inflammation and disease. The relationship between atorvastatin and ALOX15 was investigated using a rat carotid artery balloon-injury model. Hematoxylin and eosin (HE) staining showed that ALOX15 overexpression increased the thickness of the intima-media (IMT). Immunohistochemistry and western blotting showed that atorvastatin increased the expression of cellular adhesion molecules (CAMs) but decreased the expression of endothelial nitric oxide synthase (eNOS); these effects of atorvastatin were blocked by ALOX15 overexpression. In human umbilical venous endothelial cells (HUVECs), silencing of ALOX15 enhanced the effects of atorvastatin on endothelial function. Expression levels of CAMs and Akt/eNOS/NO under oxidized low-density lipoprotein (ox-LDL) stimulation were modulated by ALOX15 inhibitor and ALOX15 small interfering RNA (siRNA). Atorvastatin abolished the activation of nuclear factor-kappa B (NF-*κ*B) induced by ox-LDL. Exposure to ox-LDL induced upregulation of ALOX15 in HUVECs, but this effect was partially abolished by atorvastatin or the NF-*κ*B inhibitor pyrrolidine dithiocarbamate (PDTC). These results demonstrate that regulation of ALOX15 expression might be involved in the effects of atorvastatin on endothelial dysfunction.

## 1. Introduction

Statins are widely used to treat hyperlipidemia and prevent coronary heart disease. In addition to their lipid-lowering effects, statins produce an antiatherogenic effect by improving endothelial function, stabilizing atherosclerotic plaques, and reducing oxidative stress and endothelial inflammation [1–3]. In addition, statins enhance expression of growth factors, activate the phosphatidylinositol 3-kinase (PI3K)/Akt-mediated signaling pathway, and postpone the initiation of atherosclerosis through decreased regulation of nitric oxide (NO) synthesis by upregulating endothelial nitric oxide synthase (eNOS) mRNA expression and decreasing superoxide anion O_2_-production in endothelial cells [4, 5]. Pretreatment of endothelial cells with atorvastatin reduced expression of intracellular adhesion molecule-1 (ICAM-1), cluster of differentiation 36 (CD36), von Willebrand factor (vWF), vascular cell adhesion molecule-1 (VCAM-1), cluster of differentiation 31 (CD31), and E/P-selectin [6, 7]. Therefore, activation of the PI3K/Akt/eNOS pathway and downregulation of adhesion molecule expression might be involved in the beneficial effects of statins, but the mechanism by which statins improve endothelial function has not been fully elucidated.

Lipoxygenases (LOXs) constitute a heterogeneous family of lipid-peroxidizing enzymes that catalyze the oxidation of polyunsaturated fatty acids [8]. In mammals, LOXs are named according to the genes of the human ortholog. The human genome involves six functional LOX genes (ALOX15, ALOX15B, ALOX12, ALOX12B, ALOXE3, and ALOX5) [9, 10]. There is compelling evidence for a proinflammatory effect of ALOX15 through the formation of ox-LDL which accelerates foam cell formation and through its role in signaling of angiotensin II mediated mechanisms and vascular smooth muscle cell proliferation [11, 12]. ALOX15 and linoleic acid metabolite 13-S-hydroxyoctadecadienoic acid (13-HODE) mediate monocyte adhesion onto the blood vessel wall by activating protein kinase C (PKC) and expression of cellular adhesion molecules (CAMs) [13, 14]. Moreover, 13-HODE exerts a proinflammatory effect via transcription factors such as nuclear factor-kappa B (NF-*κ*B) [15]. ALOX15 activation is involved in the pathogenesis of atherosclerosis, hypertension, and diabetes [16]. Deletion of ALOX15 alters macrophage and islet function in nonobese diabetic ALOX15 (null) mice, leading to decreased mRNA and protein levels of proinflammatory cytokines and preventing the development of type 1 diabetes [17]. Inhibition of the initial phase of the development of atherosclerosis by statins may reduce lipid peroxidation during atherosclerotic plaque formation [18]. ALOX15 might participate in the mechanism by which statins improve endothelial function because ALOX15 is a key enzyme involved in lipid oxidation; however, little is known about the effect of atorvastatin on ALOX15.

In this paper, we identify ALOX15 as a new mediator of the effects of statins on endothelial function.

## 2. Materials and Methods

Detailed materials and methods are described in the Supplementary Material available online at http://dx.doi.org/10.1155/2016/6769032.

### 2.1. Construction of siRNA and Lentivirus Encoding ALOX15

ALOX15 shRNA lentiviral particles were purchased from Santa Cruz Biotechnology, Inc. (Santa Cruz, CA, USA). Human ALOX15 cDNA (accession number NM-001140) was subcloned into the NotI/NsiI site of pLV5-GFP (GenePharma, Shanghai, China). Rat ALOX15 cDNA (accession number NM-031010) was subcloned into the AgeI/AgeI site of pGV287-EGFP (GeneChem, Shanghai, China). The presence of both genes was confirmed by sequencing. The resulting recombinant vector was transfected into 293T cells with Lipofectamine® 2000 Plus (Invitrogen, CA, USA) in order to package viral particles expressing ALOX15. Green fluorescent protein- (GFP-) expressing lentivirus was used as a control.

### 2.2. Animal Models of Carotid Balloon Injury and Lentiviral Gene Transfer

Ten-week-old male Sprague-Dawley rats weighing 380–420 g (Animal Experiment Center of Chong Qing Medical University) were utilized. All animals were housed under conventional conditions in the animal care facility. All animals received humane care in compliance with the Principles of Laboratory Animal Care formulated by the National Society for Medical Research and the Guide for the Care and Use of Laboratory Animals. The animal experimental protocol was approved by the Animal Care Committee of The First Affiliated Hospital of Chongqing Medical University. Rats were fed standard laboratory chow and tap water ad libitum. Experiments were performed under general anesthesia achieved by an injection of ketamine (80 mg/kg) and xylazine (5 mg/kg). The rat carotid injury model was performed as described previously [19]. The left common carotid artery was denuded of endothelium by the intraluminal passage of a 2 F balloon catheter (Fogarty, Edwards Lifesciences, LLC, Irvine, CA, USA) introduced through the external carotid artery. During denudation of the endothelium, the balloon was inflated with 0.02 mL of 0.9% sodium chloride (saline) and then withdrawn through the common carotid artery to the carotid bifurcation with constant rotation. The denudation procedure was repeated two additional times to ensure complete endothelial denudation. Next, heparin (200 units/kg) was intraperitoneally injected to prevent thrombus formation. The protocol used for introducing lentiviruses into balloon-injured carotid arteries has been previously described [20]. The isolated distal segment of the injured artery from the proximal edge of omohyoid muscle to the carotid bifurcation was washed with 200 *μ*L of saline and incubated with lentivirus (1.0 × 10^8^ TU/mL) expressing either GFP or ALOX15 for 20 minutes. Seven days or fourteen days later, the balloon-injured, lentivirus-inoculated segment was perfused with saline and removed. For the atorvastatin group, atorvastatin (4 mg/kg per day) was orally administered daily to the rats for 14 days beginning shortly after the operation.

### 2.3. Morphometric Analysis of Immunohistochemistry

To assess the inflammatory process and endothelial integrity, protein expression levels of VCAM-1, ICAM-1, and eNOS were assayed by western blot analysis.

### 2.4. Cell Culture

HUVECs were purchased from Nanjing KeyGen Biotech and cultured in Roswell Park Memorial Institute- (RPMI-) 1640 medium with 10% fetal bovine serum at 37°C in an incubator containing 5% CO_2_. The fourth to eighth passages of HUVECs were used in all experiments.

### 2.5. Enzyme Immunoassay Measurement

Frozen tissue samples were homogenized for one minute on low speed in cold PBS. 13(S)-HODE from tissue samples and HUVECs were extracted by ice cold lysis buffer. An aliquot was reserved for protein determination (BCA). The organic phase of the solution was extracted using 3-fold excess of saturated ethyl acetate. Pooled organic phase solutions were dried completely under cold temperature. Residues were dissolved in 25 *μ*L of ethanol and 125 *μ*L of assay buffer (provided with EIA kits) for EIAs. 13(S)-HODE levels were measured in tissues and cells by use of a commercially available enzyme immunoassay (EIA) kit (Novus Biologicals).

### 2.6. Quantitative Real-Time Reverse-Transfection PCR

Total cellular RNA was isolated from tissue samples and HUVECs using TRIzol (Invitrogen, USA) and the reverse transcription reaction was performed with 1 *μ*g RNA in 20 *μ*L reaction using PrimeScript*™* RT reagent kit (Takara, Japan) at 42°C for 2 min and 37°C for 15 min followed by 85°C for 5 sec. The qPCR was performed on a StepOnePlus*™* Real-Time PCR System (Applied BioSystems, USA) using SYBR Green method with the following parameters: preheating for 30 sec at 95°C, followed by 45 cycles of denaturation for 5 sec at 95°C, and annealing for 34 sec at 60°C. Primers were designed using Primer-Blast (http://www.ncbi.nlm.nih.gov/tools/primer-blast/) and synthesized by Sangon Biotech (Shanghai, China). All mRNA expression levels were normalized to those of GAPDH. The primer sequences used were rat ALOX15 (forward: GTCTACTCCACCACCTATTTTC, reverse: CTGTGCTCATTGCCTTGTC) and human ALOX15 (forward: GGAGCCTTCCTAACCTACAGC, reverse: CTCACGATTCCTTCCACATACC).

### 2.7. Western Blotting Analysis

Protein expression levels of ALOX15, VCAM-1, ICAM-1, nuclear factor of kappa light polypeptide gene enhancer in B-cells inhibitor alpha (I*κ*B-*α*), NF-*κ*B-p65, Akt, p-Akt (Ser473), eNOS, and p-eNOS (Ser1177) were assayed by western blot analysis.

### 2.8. Statistical Analysis

All values are expressed as mean ± SEM. Comparisons between groups were performed using one-way analysis of variance (ANOVA) followed by the Student-Newman-Keuls (SNK) test or Dunnett's test using SPSS 17.0 software (IBM Corp., Armonk, NY, USA). *P* values less than 0.05 were considered statistically significant.

## 3. Results

### 3.1. Gene Transfer Efficacy and Effect of ALOX15 on Endothelial Proliferation* In Vivo*

**Figure 1:**
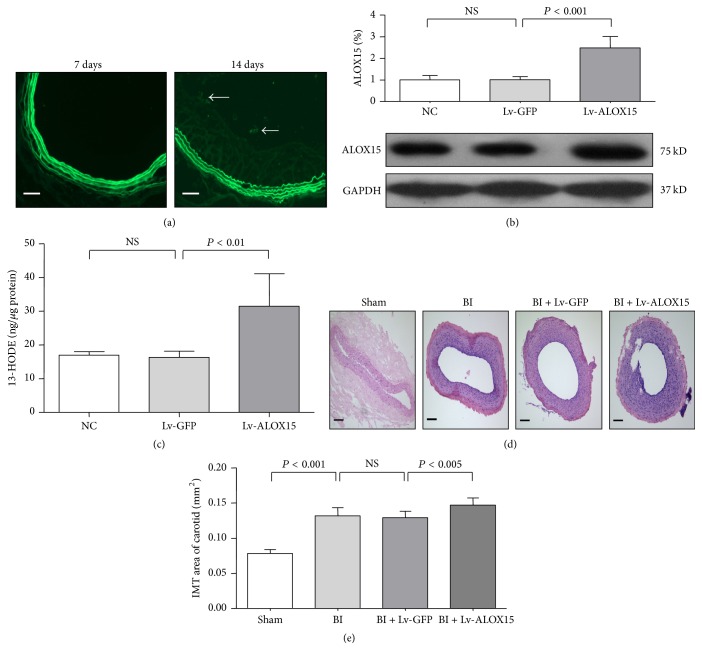
Lentivirus-based ALOX15 overexpression in the rat carotid artery and the effect of ALOX15 on neointima formation in rat balloon-injured (BI) carotid arteries. (a) Representative images showing no detectable green fluorescence after 7 days, but pronounced fluorescence after 14 days, in the neointima of carotid arteries after transfection with lentiviral constructs coexpressing GFP. (b) Western blot analyses showed that Lv-ALOX15 upregulated ALOX15 protein expression. (c) EIAs showed that Lv-ALOX15 upregulated linoleic acid metabolite 13-HODE. (d) Immediately after BI, arteries were treated with normal saline, Lv-GFP, or Lv-ALOX15. Representative photomicrographs of carotid arteries 14 days after BI. The artery cross sections were stained with HE. (e) Quantitative analysis of IMT. The arrows indicate endothelial cells expressing GFP. The scale bar represents 100 *μ*m. Results are expressed as mean ± SD. NS: not significant. *N* = 4 or 6 per group.

To assess the efficacy of* in vivo* gene transfer using GFP-conjugated lentiviral constructs, the distal segments of injured left carotid arteries were incubated with saline, Lv-GFP, or Lv-ALOX15 for 20 min immediately after the balloon injury was performed. After 7 or 14 days, the artery segments were harvested, fixed, paraffin-embedded, and sectioned. Because GFP expression provides a convenient way to test the transfection efficiency of lentivirus [21], GFP fluorescence in the carotid endothelium was examined. GFP fluorescence observations suggested successful transfection of ALOX15 at 14 days (Figure 1(a)). qRT-PCR showed no significant difference between the ALOX15 mRNA expression levels of the Lv-GFP-transduced and Lv-ALOX15-transduced vessels (Figures S1(a) and 1(b) in the online-only Supplementary Material). Western blotting showed no significant difference between the ALOX15 expression levels of the saline-treated and Lv-GFP-transduced vessels. However, ALOX15 expression was markedly increased in Lv-ALOX15-transduced vessels in comparison with that of the saline or Lv-GFP-incubated vessels (Figure 1(b), Table 1, *P* < 0.001). Enzyme immunoassays showed that 13-HODE expression was markedly increased in Lv-ALOX15-transduced vessels in comparison with that of the saline or Lv-GFP-incubated vessels (Figure 1(c), Table 1, *P* < 0.01). HE staining was used to test the effect of ALOX15 on endothelial proliferation. The distal segments of the injured left carotid arteries were harvested, fixed, paraffin-embedded, and sectioned 14 days after the balloon injury was performed. As shown in Figures 1(d) and 1(e) and Table 1, balloon injury significantly increased IMT in comparison with that of the Sham group (*P* < 0.001). Interestingly, Lv-ALOX15 significantly increased IMT in comparison with Lv-GFP (*P* < 0.005). The Lv-GFP- and saline-treated groups did not show a significant difference in IMT following balloon injury. These results indicate that the lentiviral construct system is an effective* in vivo* delivery method for inducing ALOX15 protein expression and activity in the carotid artery. Importantly, ALOX15 overexpression induced by Lv-ALOX15 enhanced endothelial proliferation in the rat carotid artery balloon injury model.

### 3.2. ALOX15 Overexpression Reduced the Protective Role of Atorvastatin* In Vivo*

**Figure 2:**
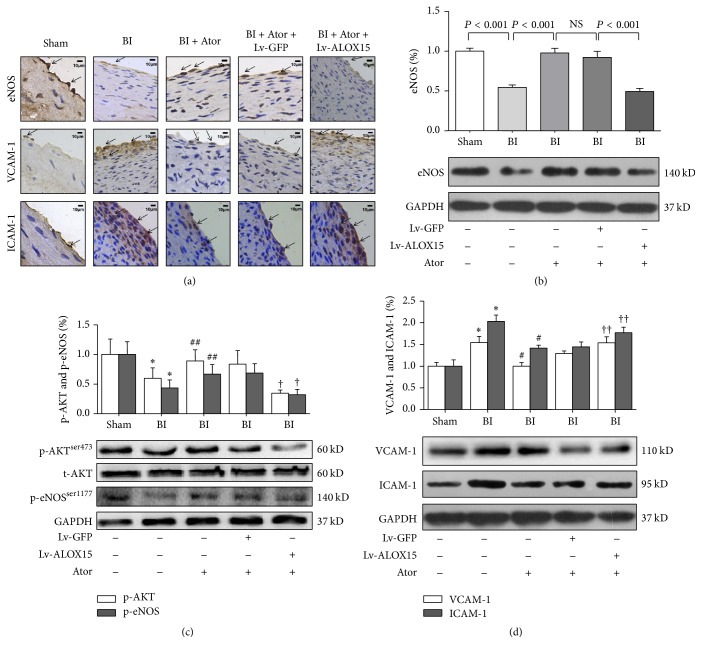
Expression levels of AKT, eNOS, VCAM-1, and ICAM-1 were modulated by lentivirus-based ALOX15 overexpression in rat balloon-injured (BI) carotid arteries 14 days after transfection. (a) Immunohistochemical analysis of eNOS, VCAM-1, and ICAM-1 in BI carotid arteries. The arrows indicate positive staining. The scale bar represents 5 *μ*m. (b) Western blot analysis of eNOS expression in BI carotid arteries. (c) Western blot analysis of p-AKT/p-eNOS expression in BI carotid arteries to assess the activity levels of AKT and eNOS. The protein level of p-eNOS was normalized against that of GAPDH because total eNOS expression was changed. (d) Western blot analysis of VCAM-1/ICAM-1 expression in BI carotid arteries. The scale bar represents 10 *μ*m. Results are expressed as mean ± SD. Ator: atorvastatin; NS: not significant. ^*∗*^ *P* < 0.01 versus the Sham group; ^#^ *P* < 0.001 and ^##^ *P* < 0.05 versus the BI group; ^†^ *P* < 0.01 and ^††^ *P* < 0.05 versus the BI + Lv-GFP group. *N* = 4 per group.

Interventional vascular procedures such as balloon angioplasty and stent placement have been shown to result in greater restenosis rates in the clinic [22]. These vascular procedures cause mechanical injury to the artery, leading to a cascade of inflammatory events and inactivation of vascular factors, but statins partially inhibit such responses of the endothelium to injury [23–25]. To test the effects of atorvastatin on endothelial injury and assess the participation of ALOX15 in such effects, we examined Akt/eNOS expression, phosphorylation of Akt (p-Akt^Ser473^) and eNOS (p-eNOS^Ser1177^), and VCAM-1/ICAM-1 in balloon-injured carotid arteries using immunohistochemistry and western blotting. As shown in Figure 2(a) and Table 2, the groups subjected to vascular balloon injury showed significantly reduced levels of total eNOS, but increased levels of VCAM-1/ICAM-1, in comparison with those of the Sham group. In the injured arteries, atorvastatin treatment significantly increased downregulation of eNOS but significantly decreased upregulation of VCAM-1/ICAM-1; however, the group treated with Lv-ALOX15 showed significantly decreased eNOS abundance, but significantly increased VCAM-1/ICAM-1 abundance, in comparison with the levels of these proteins in the group treated with Lv-GFP. Consistently, western blot analysis showed that atorvastatin treatment increased total eNOS protein expression (*P* < 0.001), as well as expression of p-Akt^Ser473^/p-eNOS^Ser1177^ (*P* < 0.05), while it decreased protein levels of VCAM-1 and ICAM-1 (*P* < 0.001). These results indicate that atorvastatin might regulate the activity of eNOS through modulating phosphorylation at these sites. Moreover, the group treated with Lv-ALOX15 showed decreased protein expression of eNOS (*P* < 0.001), p-Akt^Ser473^ (*P* < 0.001), and p-eNOS^Ser1177^ (*P* < 0.005), but significantly increased protein expression of VCAM-1 and ICAM-1 (*P* < 0.01), in comparison with the protein levels of the group treated with Lv-GFP (Figures 2(b), 2(c), and 2(d), Table 2). These results indicate that ALOX15 might be involved in the effects of atorvastatin on endothelial function* in vivo*.

### 3.3. ALOX15 Was Involved in Effects of Atorvastatin on Endothelial Function* In Vitro*

**Figure 3:**
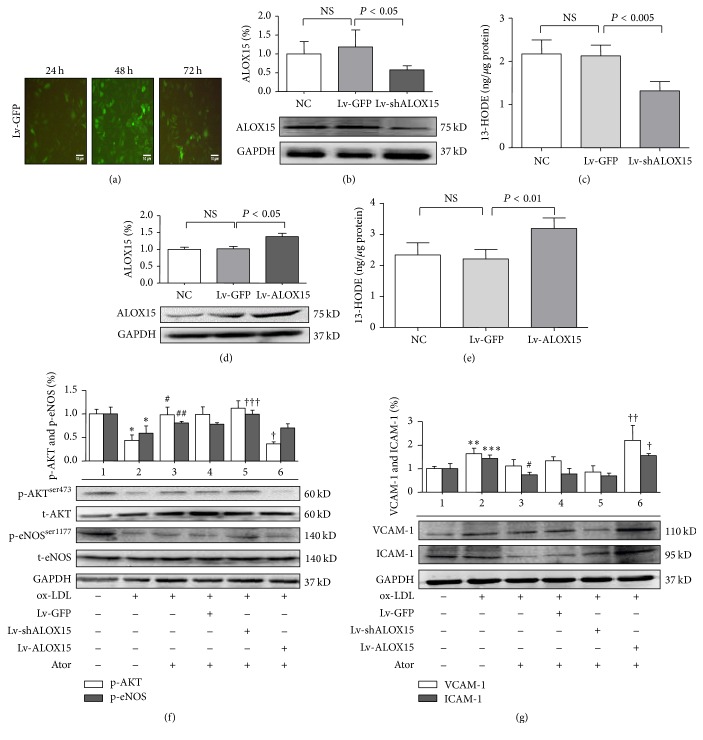
Changes in expression levels of ALOX15, AKT, eNOS, VCAM-1, and ICAM-1 in HUVECs 24 h following exposure to ox-LDL (100 mg/L) or atorvastatin (10 *μ*M). (a) Photographs of Lv-GFP-transfected HUVECs. GFP expression was visualized by fluorescence microscopy (100x) at 24, 48, and 72 h after transfection. The scale bar represents 10 *μ*m. (b) and (c) Western blot and EIAs indicated that Lv-shALOX15 downregulated ALOX15 protein expression and enzyme activity. (d) and (e) Western blot and EIAs indicated that Lv-ALOX15 upregulated ALOX15 protein expression and enzyme activity. (f) Western blot analysis of AKT/eNOS expression in HUVECs. (g) Western blot analysis of VCAM-1/ICAM-1 expression in HUVECs. Group 1: normal control group; Group 2: ox-LDL (100 mg/L); Group 3: ox-LDL (100 mg/L) + atorvastatin (10 *μ*M); Group 4: Lv-GFP + ox-LDL (100 mg/L) + atorvastatin (10 *μ*M); Group 5: Lv-shALOX15 + ox-LDL (100 mg/L) + atorvastatin (10 *μ*M); and Group 6: Lv-shALOX15 + ox-LDL (100 mg/L) + atorvastatin (10 *μ*M). Results are expressed as mean ± SD. NC: normal control group; NS: not significant. ^*∗*^ *P* < 0.001, ^*∗∗*^ *P* < 0.01, and ^*∗∗∗*^ *P* < 0.05 versus Group 1; ^#^ *P* < 0.001 and ^##^ *P* < 0.05 versus Group 2; and ^†^ *P* < 0.001, ^††^ *P* < 0.01, and ^†††^ *P* < 0.05 versus Group 4. *N* = 4 per group.

Atherosclerosis (AS) is characterized by chronic oxidative stress and inflammatory changes in vascular tissue. Increased local and systemic levels of ox-LDL induce endothelial cell activation, dysfunction, apoptosis, and impaired vasorelaxation, which contribute to the development and progression of atherosclerosis [26, 27]. To further assess the role of ALOX15 in the effects of atorvastatin on endothelial function* in vitro*, an ox-LDL-stimulated endothelial injury model was established in HUVECs. The MTT assay was performed after HUVECs were exposed to various concentrations of ox-LDL (50, 100, and 150 mg/L) for 24 h. ox-LDL damaged HUVECs in a concentration-dependent manner. After exposure to 50 mg/L and 100 mg/L ox-LDL, cell viability was reduced to 93.8% (*P* > 0.05) and 75.8% (*P* < 0.001), respectively, of that of the control group, whereas exposure to 150 mg/L ox-LDL reduced cell viability to 41.5% (*P* < 0.001) of that of the control group (Figure S1(c) in the online-only Supplementary Material). To assess the effects of Lv-shALOX15 and Lv-ALOX15* in vitro*, GFP expression was visualized by fluorescence microscopy at 24, 48, and 72 h after transfection (Figure 3(a), Table 3), whereas transfection efficiency was determined by flow cytometry (Figure S2 in the online-only Supplementary Material). The cells treated with Lv-shALOX15 showed significantly reduced ALOX15 protein and 13-HODE expression in comparison with that of the Lv-GFP group (Figures 3(b) and 3(c), Table 3, *P* < 0.05), while the cells treated with Lv-ALOX15 showed significantly upregulated ALOX15 protein and 13-HODE expression in comparison with that of the Lv-GFP group (Figures 3(d) and 3(e) and Table 3, *P* < 0.01). However, ALOX15 mRNA was altered by Lv-shALOX15 but not Lv-ALOX15 (Figures S3(a)–3(d) in the online-only Supplementary Material). These results suggest that both ALOX15 protein expression and enzyme activity were altered after transfection with Lv-shALOX15 or Lv-ALOX15. As shown in Figures 3(f) and 3(g) and Table 3, changes in total Akt and total eNOS protein levels were not observed in the Lv-shALOX15 and Lv-ALOX15 groups. ox-LDL stimulation reduced protein levels of p-Akt^Ser473^ and p-eNOS^Ser1177^ (*P* < 0.001) but upregulated protein expression levels of VCAM-1 and ICAM-1 (*P* < 0.05), in comparison with the protein levels of the NC group. Atorvastatin treatment increased protein expression of p-Akt^Ser473^ and p-eNOS^Ser1177^ (*P* < 0.05) but slightly decreased protein expression of VCAM-1 (no significant difference) and significantly decreased protein expression of ICAM-1 (*P* < 0.001), in comparison with the protein levels of the ox-LDL group. Silencing of ALOX15 slightly increased protein expression of p-Akt^Ser473^ (no significant difference) and increased protein expression of p-eNOS^Ser1177^ (*P* < 0.05) but slightly decreased protein expression levels of VCAM-1 and ICAM-1 (no significant difference), in comparison with the protein levels of the Lv-GFP group. Interestingly, Lv-ALOX15 significantly decreased protein expression of p-Akt^Ser473^ (*P* < 0.001) and slightly decreased protein expression of p-eNOS^Ser1177^ (no significant difference) but significantly increased protein expression levels of VCAM-1 and ICAM-1 (*P* < 0.01), in comparison with the protein levels of the Lv-GFP group. These results indicate that atorvastatin significantly increased protein levels of p-Akt^Ser473^ and p-eNOS^Ser1177^ but decreased protein levels of adhesion molecules in HUVECs. Silencing of ALOX15 enhanced the effects of atorvastatin on protein levels of Akt, eNOS, and CAMs* in vitro*, whereas overexpression of ALOX15 attenuated these effects.

### 3.4. 15-LOX-1 Is Required for Upregulation of CAMs Expression and Inhibition of PI3K-Akt-eNOS Signaling under ox-LDL Stimulation* In Vitro*

**Figure 4:**
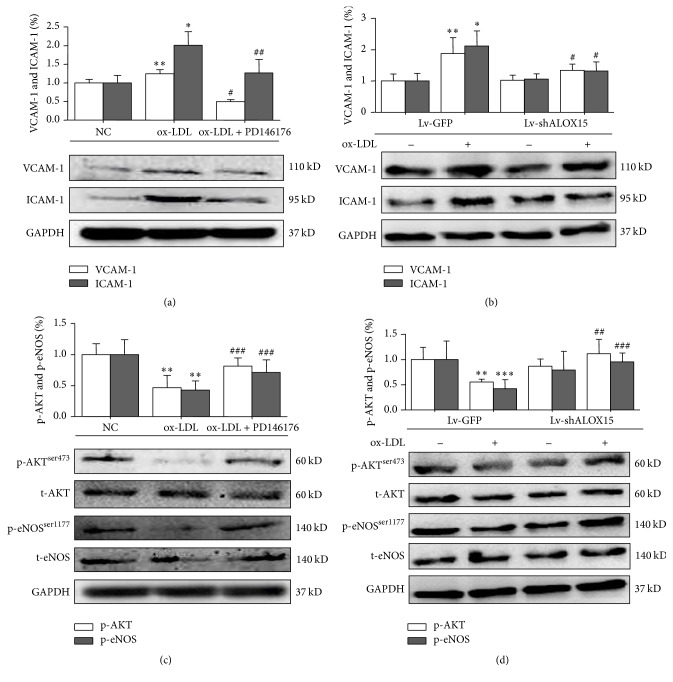
Effects of PD146176 (20 *μ*M) and Lv-shALOX15 on expression levels of VCAM-1, ICAM-1, AKT, and eNOS in HUVECs. (a) and (b) Western blot analysis of VCAM-1/ICAM-1 in HUVECs. (c) and (d) Western blot analysis of AKT/eNOS in HUVECs. Results are expressed as mean ± SD. NC: normal control group. ^*∗*^ *P* < 0.001, ^*∗∗*^ *P* < 0.01, and ^*∗∗∗*^ *P* < 0.05 versus the NC group; ^#^ *P* < 0.001, ^##^ *P* < 0.01, and ^###^ *P* < 0.01 versus the ox-LDL group or ox-LDL + Lv-GFP group. *N* = 5 per group.

We next assessed the relationship between ALOX15, CAMs expression, and the PI3K-Akt-eNOS signaling cascade under ox-LDL stimulation. Expression levels of VCAM-1 and ICAM-1 (Figure 4(a) and Table 4, *P* < 0.001; Figure 4(b) and Table 4, *P* < 0.01) were significantly increased in ox-LDL-stimulated HUVECs, but those of p-Akt^Ser473^ and p-eNOS^Ser1177^ (Figure 4(c) and Table 4, *P* < 0.005; Figure 4(d) and Table 4, *P* < 0.05) significantly decreased. In HUVECs, 15-LOX-1 inhibitor PD-146176 abolished ox-LDL induced upregulation of VCAM-1 and ICAM-1 (Figure 4(a) and Table 4, *P* < 0.001), as well as downregulation of p-Akt^Ser473^ and p-eNOS^Ser1177^ (Figure 4(c) and Table 4, *P* < 0.05). Lv-shALOX15 blocked expression of VCAM-1/ICAM-1 (Figure 4(b) and Table 4, *P* < 0.001) under ox-LDL stimulation but activated expression of p-Akt^Ser473^ and p-eNOS^Ser1177^ (Figure 4(d) and Table 4, *P* < 0.05), in comparison with the levels of the Lv-GFP group. These results suggest that ALOX15 is essential for ox-LDL induced enhancement of CAMs expression and inactivation of the PI3K-Akt-eNOS signaling cascade* in vitro*.

### 3.5. Atorvastatin Reduced 15-LOX-1 Expression via NF-*κ*B

**Figure 5:**
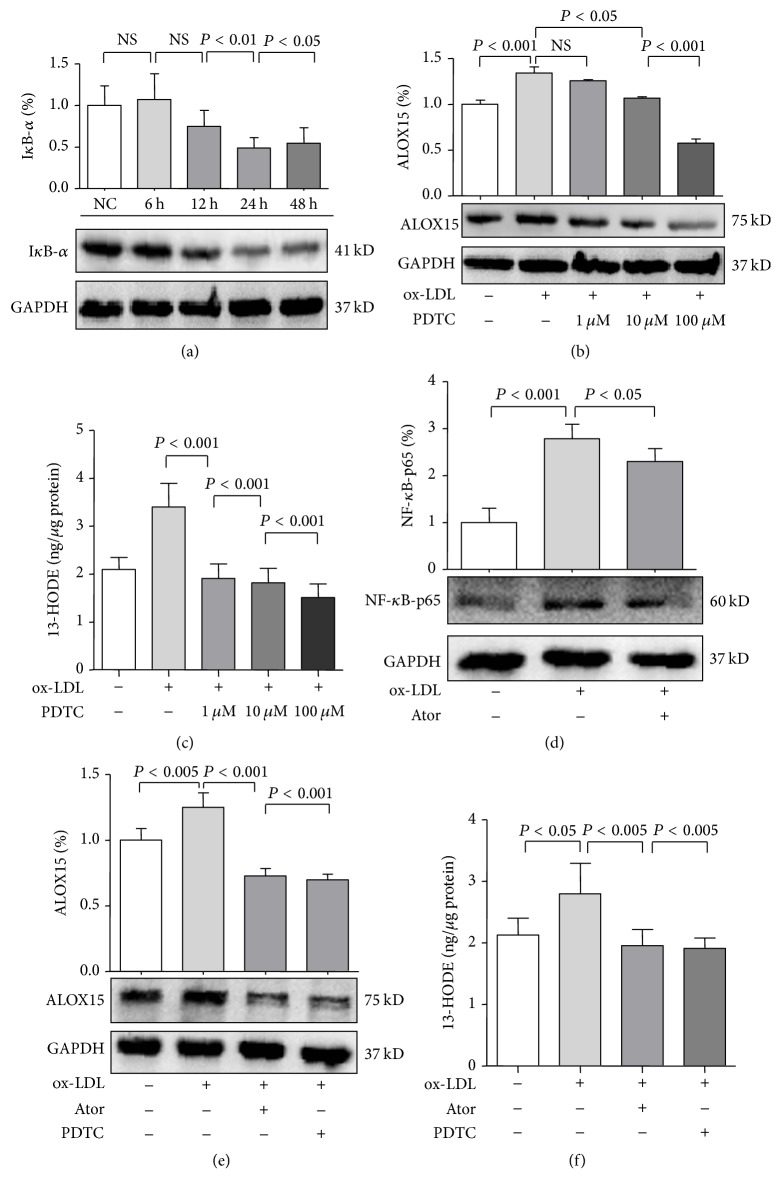
The relationship between atorvastatin and ALOX15. (a) ox-LDL (100 mg/L) increased NF-*κ*B activity in a time-dependent manner. (b) and (c) NF-*κ*B activity affected ox-LDL-induced expression of ALOX15 in HUVECs, which were preincubated with pyrrolidine dithiocarbamate (PDTC) (1 *μ*M to 100 *μ*M) for 1 h before ox-LDL stimulation. (d) HUVECs were preincubated with atorvastatin (10 *μ*M) for 8 h before ox-LDL stimulation, after which NF-*κ*B-p65 protein levels were examined by western blot analysis. (e) and (f) Effects of atorvastatin and PDTC on ALOX15 protein expression and enzyme activity in HUVECs. Results are expressed as mean ± SD. NC: normal control; NS: not significant. *N* = 3 or 4 per group.

In endothelial cells, ox-LDL activates diverse secondary messengers, including NF-*κ*B and AP-1, two oxidative stress-responsive transcription factors important in the regulation of cytokines, chemokines, and adhesion molecules [28]. As shown in Figure 5(a) and Table 5, the apparent degradation of I*κ*B-*α* in HUVECs in a time-dependent manner under ox-LDL stimulation suggested enhanced NF-*κ*B activity (24 h, *P* < 0.01; 48 h, *P* < 0.05). As both 5-lipoxygenase and 12-lipoxygenase were identified to be transcriptionally regulated by NF-*κ*B [29], we measured ALOX15 expression in HUVECs under ox-LDL stimulation. To assess the role of NF-*κ*B in regulation of LOX expression under ox-LDL stimulation, HUVECs were exposed to NF-*κ*B inhibitor pyrrolidine dithiocarbamate (PDTC) at several concentrations. After 24 h of ox-LDL stimulation, ALOX15 protein and 13-HODE expression were significantly increased in comparison with those of the control group, but this effect was largely abolished by 10 *μ*M PDTC (*P* < 0.05) and 100 *μ*M PDTC (*P* < 0.001) (Figures 5(b) and 5(c) and Table 5). Consistently, qRT-PCR showed that PDTC treatment decreased ALOX15 mRNA expression (Figures S4(a) and 4(b) in the online-only Supplementary Material), suggesting that NF-*κ*B is an important mediator of ox-LDL-stimulated ALOX15 upregulation. As shown in Figure 5(d) and Table 5, atorvastatin treatment significantly reduced expression of NF-*κ*B-p65 (*P* < 0.05) in ox-LDL-stimulated HUVECs. In addition, ox-LDL-induced ALOX15 protein and 13-HODE expression were inhibited by treatment with atorvastatin (*P* < 0.005) or PDTC (*P* < 0.005) (Figures 5(e) and 5(f) and Table 5). In addition, qRT-PCR showed that PDTC or atorvastatin treatment decreased ALOX15 mRNA expression (Figures S4(c) and 4(d) in the online-only Supplementary Material). These results suggest that inhibition of NF-*κ*B activation plays a role in the effects of atorvastatin on ALOX15 expression.

## 4. Discussion

In the present study, overexpression of ALOX15 in balloon-injured rat carotid arteries increased IMT. Atorvastatin treatment suppressed upregulation of adhesion molecules and downregulation of eNOS induced by balloon injury, whereas overexpression of ALOX15 partially eliminated these effects. Atorvastatin suppressed upregulation of adhesion molecules and downregulation of p-Akt^ser473^ and p-eNOS^ser1177^ induced by ox-LDL stimulation* in vitro*. Silencing of ALOX15 enhanced these effects of atorvastatin on adhesion molecules, Akt, and eNOS, whereas overexpression of ALOX15 eliminated these effects. ALOX15 was required for upregulation of CAMs expression and downregulation of p-Akt^ser473^ and p-eNOS^ser1177^ expression under ox-LDL stimulation. NF-*κ*B was essential for expression of ALOX15 induced by ox-LDL. Atorvastatin might reduce expression of ALOX15 via NF-*κ*B. To the best of our knowledge, these results provide the evidence that statin treatment might modulate CAMs expression and the PI3K-Akt-eNOS signaling cascade by inactivating ALOX15. These results suggest that ALOX15 is involved in the effect of atorvastatin on endothelial function.

### 4.1. Relationship between ALOX15 and the Effect of Atorvastatin on Endothelial Function

On account of minor human ALOX15 mRNA that were detected in atherosclerotic lesions, ALOX15 may play a role in the initiation of atherosclerosis but not in later stages of atherogenesis [9]. Our study demonstrates that ALOX15 overexpression markedly increases IMT, suggesting that ALOX15 plays a critical role in endothelial proliferation after arterial injury. After observing the detrimental effects of ALOX15 on the injured vessel segment, we investigated the relationship between ALOX15 and atorvastatin. In the atorvastatin-treated rats, protein levels of eNOS, p-Akt^ser473^, and p-eNOS^ser1177^ were increased, but protein levels of VCAM-1 and ICAM-1 were decreased. Overexpression of ALOX15 inhibited the beneficial effects of atorvastatin on eNOS, p-Akt^ser473^, p-eNOS^ser1177^, VCAM-1, and ICAM-1. The observed upregulation of eNOS and downregulation of VCAM-1 and ICAM-1 suggest that atorvastatin may have favorable effects on endothelial function and inflammation, confirming previous studies [30]. Consistent results were observed* in vitro*, where silencing of ALOX15 enhanced the effects of atorvastatin on endothelial function. We demonstrate here that the effects of atorvastatin on vascular endothelial function and inflammation are associated with regulation of ALOX15 expression.

### 4.2. Effect of ALOX15/ox-LDL on Adhesion Molecules and PI3K-Akt-eNOS Signaling

In human endothelial cells, ox-LDL increased expression of VCAM-1 and ICAM-1 but reduced expression of p-Akt^ser473^ and p-eNOS^ser1177^. The stimulatory effects of ox-LDL were observed at the protein level in HUVECs; interestingly, these effects were suppressed by ALOX15 inhibitors or ALOX15-shRNA. Emerging evidence suggests that ox-LDL exposure is associated with upregulation of ICAM-1 and VCAM-1, as well as inactivation of the PI3K-Akt-eNOS signaling cascade in multiple cell types; however, the mechanism by which adhesion molecules and Akt/eNOS are regulated in ox-LDL-induced endothelial dysfunction has not been fully elucidated [31–33]. The present study indicates that ALOX15 is involved in regulation of CAMs expression and the PI3K-Akt-eNOS signaling cascade in HUVECs under ox-LDL stimulation.

### 4.3. Role of NF-*κ*B in ox-LDL-Induced Upregulation of ALOX15

Previous studies have demonstrated that ox-LDL mainly activates NF-*κ*B, an oxidative stress-responsive transcription factor important in the regulation of cytokines, chemokines, and inflammatory molecules in endothelial cells, including 5-lipoxygenase and 12-lipoxygenase [29, 34, 35]. Although our work, similar to previous works, showed reduced I*κ*B-*α* activity in HUVECs under ox-LDL stimulation, we also demonstrated that ox-LDL induced upregulation of ALOX15 and NF-*κ*B, which was necessary for ox-LDL-induced ALOX15 expression. Moreover, atorvastatin inhibited overexpression of NF-*κ*B and ALOX15 induced by ox-LDL stimulation in HUVECs, while inhibitors of atorvastatin and NF-*κ*B had similar effects on ALOX15 expression. A large number of proinflammatory genes were modulated by NF-*κ*B, which can be activated by ox-LDL. Therefore, NF-*κ*B activation might be involved in ox-LDL-induced upregulation of VCAM-1 and ICAM-1 and downregulation of Akt and eNOS. The role of statins as inhibitors of NF-*κ*B has also been investigated in cellular and animal models. Exposure of vascular smooth muscle cells (VSMCs) to atorvastatin prevents TNF-*α* and angiotensin II-induced activation of NF-*κ*B and subsequent upregulation of inflammatory mediators [36]. Simvastatin and atorvastatin attenuate ox-LDL-induced activation of NF-*κ*B in human coronary artery endothelium cells [37]. In the present paper, ox-LDL led to regulation of CAMs and eNOS; then these effects were inhibited by blocking ALOX15. Actually, we observed similar phenomenon by blocking ALOX15; hence we considered ox-LDL as a stimulation factor to study the relationship between ALOX15 and atorvastatin step by step. Simvastatin treatment reduces NF-*κ*B activity in atherosclerotic plaques and circulating mononuclear cells in animals with diet-induced atherosclerosis independently of its lipid-lowering effects [38]. In agreement with previous studies, the present study indicates that atorvastatin treatment reduces NF-*κ*B expression. In addition, inhibition of NF-*κ*B induced by treatment with atorvastatin might decrease the activity of ALOX15.

## Supplementary Material

The Supplementary Material is the gating strategy for detailed materials and methods, the detection of cell viability by MTT assay, the detection of transfection efficiency by Flow cytometry analysis and the detection of ALOX15 mRNA by qRT-PCR analysis.

## Figures and Tables

**Table 1:** Numbers in results.

Figure 1	Group	Mean ± SD
(b)	Lv-ALOX15	2.479 ± 0.526

(c)	Lv-ALOX15	31.528 ± 9.632

(e)	BI	0.132 ± 0.012
	BI + Lv-ALOX15	0.147 ± 0.010

**Table 2:** Numbers in results.

Figure 2	Group	Mean ± SD
(b)	BI + Ator	0.977 ± 0.059
	BI + Ator + ALOX15	0.494 ± 0.038

(c) p-AKT	BI + Ator	0.889 ± 0.192
	BI + Ator + ALOX15	0.344 ± 0.054

(c) p-eNOS	BI + Ator	0.667 ± 0.161
	BI + Ator + ALOX15	0.319 ± 0.092

(d) VCAM-1	BI + Ator	1.002 ± 0.183
	BI + Ator + ALOX15	1.538 ± 0.138

(d) ICAM-1	BI + Ator	1.417 ± 0.067
	BI + Ator + ALOX15	1.770 ± 0.128

**Table 3:** Numbers in results.

Figure 3	Group	Mean ± SD
(b)	Lv-shALOX15	0.577 ± 0.111

(c)	Lv-shALOX15	1.315 ± 0.215

(d)	Lv-ALOX15	1.380 ± 0.097

(e)	Lv-ALOX15	3.192 ± 0.336

(f) p-AKT	ox-LDL	0.441 ± 0.112
	ox-LDL + Ator	0.981 ± 0.163
	ox-LDL + Ator + Lv-ALOX15	0.365 ± 0.041

(f) p-eNOS	ox-LDL	0.591 ± 0.155
	ox-LDL + Ator	0.809 ± 0.032
	ox-LDL + Ator + Lv-shALOX15	0.994 ± 0.084

(g) VCAM-1	ox-LDL	1.635 ± 0.239
	ox-LDL + Ator + Lv-ALOX15	2.206 ± 0.633

(g) ICAM-1	ox-LDL	1.435 ± 0.152
	ox-LDL + Ator	0.740 ± 0.111
	ox-LDL + Ator + Lv-ALOX15	1.553 ± 0.089

**Table 4:** Numbers in results.

Figure 4	Group	Mean ± SD
(a) VCAM-1	ox-LDL	1.249 ± 0.111
	ox-LDL + PD146176	0.498 ± 0.056

(a) ICAM-1	ox-LDL	2.012 ± 0.362
	ox-LDL + PD146176	1.267 ± 0.362

(b) VCAM-1	ox-LDL + Lv-GFP	1.118 ± 0.078
	ox-LDL + Lv-shALOX15	0.916 ± 0.016

(b) ICAM-1	ox-LDL + Lv-GFP	1.437 ± 0.147
	ox-LDL + Lv-shALOX15	0.632 ± 0.056

(c) p-AKT	ox-LDL	0.465 ± 0.201
	ox-LDL + PD146176	0.814 ± 0.134

(c) p-eNOS	ox-LDL	0.428 ± 0.145
	ox-LDL + PD146176	0.711 ± 0.203

(d) p-AKT	ox-LDL + Lv-GFP	0.550 ± 0.058

(d) p-eNOS	ox-LDL + Lv-shALOX15	1.113 ± 0.289
	ox-LDL + Lv-GFP	0.422 ± 0.174
	ox-LDL + Lv-shALOX15	0.953 ± 0.177

**Table 5:** Numbers in results.

Figure 5	Group	Mean ± SD
(a)	24 h	0.491 ± 0.123
	48 h	0.545 ± 0.187

(b)	ox-LDL + PD146176 (10 *μ*M)	1.068 ± 0.013
	ox-LDL + PD146176 (100 *μ*M)	0.576 ± 0.045

(c)	ox-LDL	3.401 ± 0.489
	ox-LDL + PD146176 (1 *μ*M)	1.192 ± 0.297
	ox-LDL + PD146176 (100 *μ*M)	1.819 ± 0.298
	ox-LDL + PD146176 (100 *μ*M)	1.510 ± 0.285

(d)	ox-LDL + Ator	2.303 ± 0.274

(e)	ox-LDL + Ator	0.727 ± 0.056
	ox-LDL + PD146176	0.698 ± 0.042

(f)	ox-LDL	2.803 ± 0.492
	ox-LDL + Ator	1.955 ± 0.264
	ox-LDL + PD146176	1.911 ± 0.172

## Additional Points

*Limitations of the Present Study*. The present study demonstrates that atorvastatin reduces inflammation and restores function in endothelial cells via a process involving modulation of CAMs expression and the PI3K/Akt/eNOS signaling cascade by NF-*κ*B and ALOX15. However, according to previous expression studies, levels of ALOX15 mRNA are only present in small amounts in advanced atherosclerotic lesions. Thus, despite the discovery of the interaction between ALOX15 and atorvastatin, it is unclear whether or not atorvastatin effects in the present study can indeed explain the beneficial cardiovascular effects of statins in humans. Further study is still needed to clarify this novel pathway. In summary, the results of this study provide insight into the initial pathogenesis of AS and guidance for the development of new therapeutic strategies for the treatment of AS.
