# Supplementary material for: 15-Lipoxygenase-1 Is Involved in the Effects of Atorvastatin on Endothelial Dysfunction
Source: Mediators Inflamm. 2016 Aug 10;2016:6769032. doi: 10.1155/2016/6769032 (PMC4995339; doi:10.1155/2016/6769032)

15-Lipoxygenase-1 is involved in the effects of atorvastatin on endothelial dysfunction

Peng Zhang, Xin Xing, Chunxiao Hu, Hui Yu, Qian Dong, Guanglei Chang, Shu Qin, Jian Liu, Dongying Zhang

Materials and Methods

Reagents and Antibodies

Atorvastatin was obtained from Pfizer. Ox-LDL was obtained from Beijing Union Biology. PD146176 and PDTC were purchased from Sigma Aldrich. Mouse antibodies against ALOX15, VCAM-1, and ICAM-1 were purchased from Abcam. Rabbit antibodies against IκB-α, NF-κB-p65, AKT, p-AKT (Ser473), eNOS, and p-eNOS (Ser1177) were purchased from Cell Signaling Technology. Rabbit antibodies against GAPDH were purchased from Santa Cruz.

Morphometric analysis of intimal-media thickening/immunohistochemistry

Arteries were perfusion-fixed *in situ* with 150 mL of 4% formaldehyde solution (pH 7.4) prior to morphometric analysis. Carotid arteries were sectioned (5 μm) and stained with hematoxylin and eosin. Sections were evaluated blindly using computer-assisted morphometry (Olympus Mikroskopie Hamburg, Germany and Image Pro). The thickness of the intima-media was calculated. To assess the inflammatory process and endothelial integrity, serial sections were stained with anti-VCAM-1, anti-ICAM-1, and anti-eNOS antibodies. Morphometric analyses were performed in a double-blinded manner.

Cell viability assay

Cell viability was assessed with a 3-(4,5-dimethylthiazol-2-yl)-2,5-diphenyltetrazolium bromide (MTT) cell proliferation and cytotoxicity assay kit (Beyotime Institute of Biotechnology, China) according to the manufacturer's instructions. Cells were seeded in a 96-well plate at a density of 1×10^4 cells/well and incubated for 24 h. HUVECs were exposed to ox-LDL (0, 50, 100, or 150 mg/L) for 24 h, after which the cells were placed in fresh media and MTT solution (10 μL) for 4 h, followed by incubation with formazan solution (10 μL) for 4 h at 37 °C. Optical density (OD) values at 570 nm were measured using a microplate reader (Multiskan MK33, Thermo Labsystems, Finland).

Cell transfection

Transfection of ALOX15 was achieved by lentivirus-shRNA-ALOX15 or lentivirus-ALOX15 (MOI = 100). Cells cultured with lentivirus-GFP (MOI = 100) were used as a control group. After transfection (24 h, 48 h, or 72 h), HUVECs were washed briefly with phosphate-buffered saline (PBS), released from the substrate surface with 0.25% trypsin-EDTA (Invitrogen, USA), and centrifuged at 600 × *g* for 5 minutes. Finally, the cells were filtered through a FacsCalibur cell sorter (BD FACS vantage SE, Beckman Coulter, USA).

Western blotting analysis

Cells and carotid arteries were washed with PBS and lysed using radio-immunoprecipitation assay buffer. Protein concentrations were determined using the bicinchoninic acid assay. Protein samples were denatured using sodium dodecyl sulfate polyacrylamide gel electrophoresis sample loading buffer (Beyotime Institute

of Biotechnology). An equal amount of each sample (60 µg) was subjected to electrophoresis on a 6–12% SDS-containing gel (Beyotime Institute of Biotechnology) and transferred to a polyvinylidene difluoride membrane (0.45 µm). The membranes were blocked with 5% bovine serum albumin in Tris-buffered saline solution containing 0.2% Tween-20 (0.2 M Tris base, 1.5 M NaCl, pH 7.6) for 1 h at room temperature. The membranes were incubated overnight at 4 °C with anti-ALOX15 (1:500, Abcam), anti-VCAM-1 (1:5000), anti-ICAM-1 (1:3000), anti-IκB-α (1:1000), anti-NF-κB-p65 (1:1000), anti-AKT (1:1000), anti-p-AKT (Ser473) (1:1000), anti-eNOS (1:1000), anti-p-eNOS (Ser1177) (1:1000), or anti-GAPDH (1:1000) antibodies. After washing the membranes in TBST three times, they were incubated with horseradish peroxidase (HRP)-conjugated anti-mouse (1:10000) or anti-rabbit secondary antibodies (1:10000) for 1 h at 37 °C, washed three times with TBST, and developed using an electrochemiluminescence (ECL) kit (BeyoECL Star, Beyotime Institute of Biotechnology, ShangHai, China). The autoradiographic films were scanned, after which quantitative analysis of the detected peptides was performed by densitometry using Quantity One software (Bio-Rad, Hercules, CA, USA).

Legends to Supplemental Figures

Figure S1: ALOX15 mRNA expression in the rat carotid artery after tansfection with Lv-ALOX15 and cell viability of HUVECs. A-B: qRT-PCR showed no significant difference between the ALOX15 mRNA expression levels of the Lv-GFP-transduced and Lv-ALOX15-transduced vessels. C: Ox-LDL damaged HUVECs in a concentration-dependent manner. Results are expressed as mean ± SD. N.S., not significant. N = 3 or 6 per group.

Figure S2 A-D: Flow cytometry detection of transfection efficiency. M2 stands for the GFP (+) cells.

Figure S3: ALOX15 mRNA expression in the HUVECs after tansfection with Lv-shALOX15 or Lv-ALOX15. A-B: The cells treated with Lv-shALOX15 showed significantly down-regulated ALOX15 mRNA expression in comparison with that of the Lv-GFP group. C-D: No significant difference between the ALOX15 mRNA expression levels of the Lv-GFP-transduced and Lv-ALOX15-transduced cells. Results are expressed as mean ± SD. N.S., not significant. N = 3 per group.

Figure S4: ALOX15 mRNA expression in the HUVECs after tansfection with Lv-shALOX15 or Lv-ALOX15. A-B: After 24 h of ox-LDL stimulation, ALOX15 mRNA expression were significantly increased in comparison with that of the control group, but this effect was abolished by 1μM PDTC, 10 μM PDTC and 100 μM PDTC. C-D: ox-LDL-induced ALOX15 mRNA expression were inhibited by treatment with atorvastatin or PDTC. Results are expressed as mean ± SD. N.S., not significant. N = 3 per group.

Figure S1

A

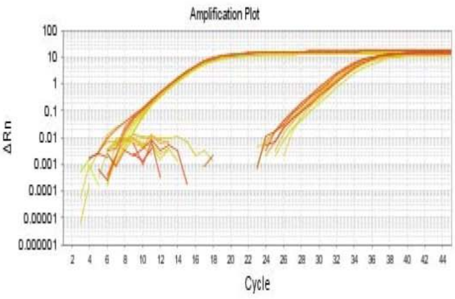

B

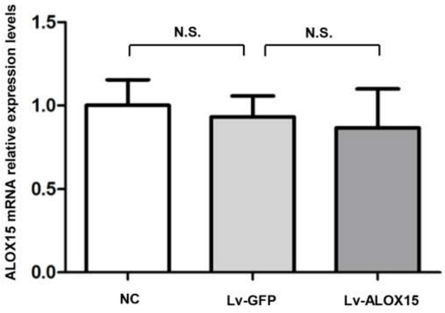

C

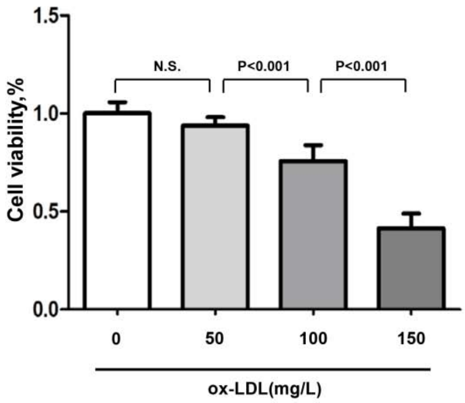

Figure S2

A

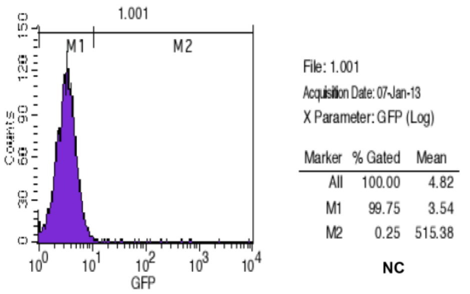

B

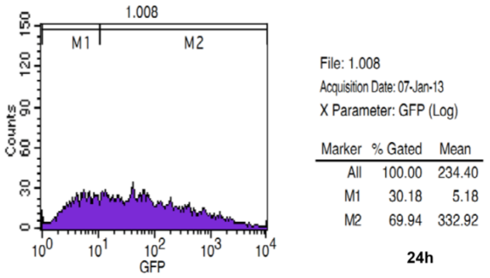

C

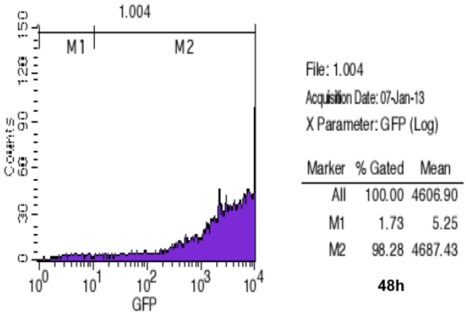

D

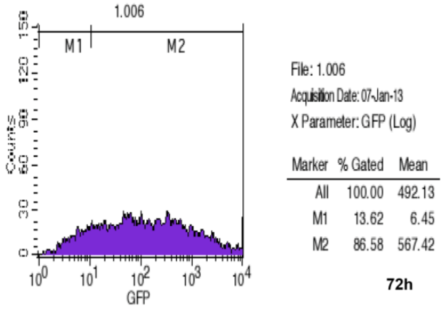

Figure S3

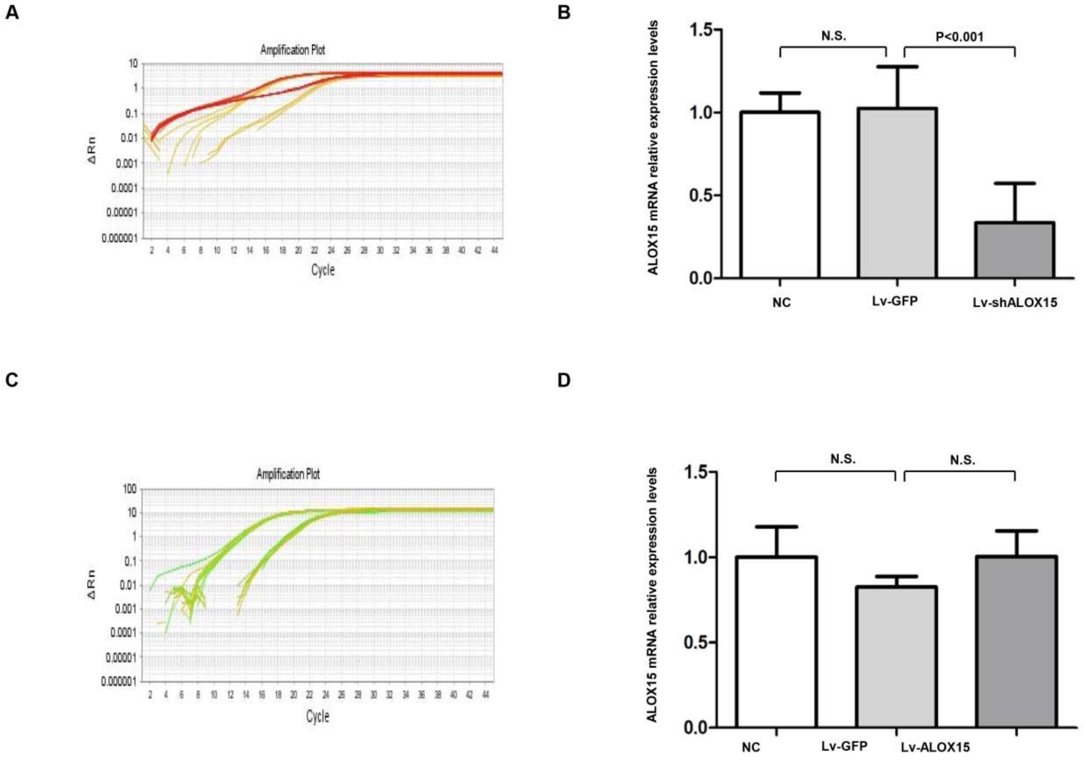

Figure S4

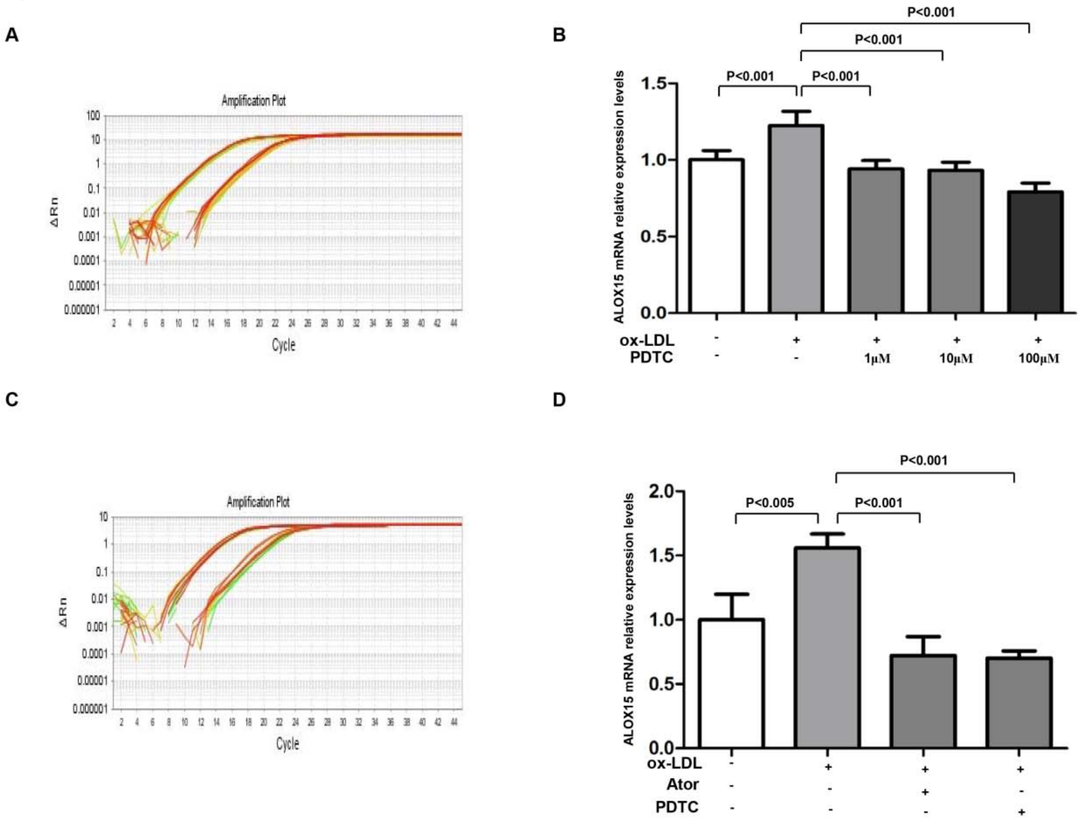

Supplement: Supplementary file 1 — The Supplementary Material is the gating strategy for detailed materials and methods, the detection of cell viability by MTT assay, the detection of transfection efficiency by Flow cytometry analysis and the detection of ALOX15 mRNA by qRT-PCR analysis. [file 6769032.f1.pdf]
